# Faecal shedding of SARS-CoV-2 from patients with asymptomatic and mild COVID-19 without gastrointestinal symptoms in Ghana

**DOI:** 10.1186/s13104-024-06790-z

**Published:** 2024-05-10

**Authors:** Enoch Aninagyei, Reuben Ayivor-Djanie, Jones Gyamfi, Theodore Owuani, Selassie Louis Ameke, Grace Semabia Kpeli, Hubert Kwame Agbogli, Priscilla Essandoh, Kwabena Obeng Duedu

**Affiliations:** 1 Department of Biomedical Sciences, School of Basic and Biomedical Sciences, University of Health and Allied Sciences, Ho, Ghana; 2 UHAS COVID-19 Centre, University of Health and Allied Sciences, Ho, Ghana; 3 West African Centre for Cell Biology of Infectious Pathogens (WACCBIP), University of Ghana, Legon, Ghana; 4 Department of Medical Laboratory Sciences, School of Allied Health Sciences, University of Health and Allied Sciences, Ho, Ghana; 5 Laboratory Department, Ghana Health Service, Ho Municipal Hospital, Ho, Ghana; 6 College of Life Sciences, Birmingham City University, City South Campus, Birmingham, UK

**Keywords:** COVID-19, SARS-CoV-2, Faecal shedding, Gastrointestinal symptoms, Sewage

## Abstract

**Objective:**

In this study, we sought to determine whether faecal shedding occurs among SARS-COV-2 positive Ghanaians, as reported elsewhere. Hence we assayed for SARS-COV-2 in the stools of 48 SARS-COV-2 confirmed patients at the Ho Municipal Hospital in Ghana.

**Results:**

Of the 48 COVID-19 patients, 45 (93.8%) had positive tests for SARS-CoV-2 faecal shedding. About 60% reported no respiratory symptoms, while only 2% (1 patient) reported gastrointestinal (GI) symptoms in the form of nausea**.** Other symptoms reported included headache (57.9%), weakness (57.9%), cough (52.6%), blocked/runny nose (47.4%), fever (31.6%), sore throat (31.6%), and shortness of breath (21.1%). One person complained of nausea (5.3%) Semi-quantitative comparison of the SARS COV-2 viral loads in matched respiratory and faecal samples using the cycle threshold (CT) values revealed no statistical differences. Furthermore, the duration between collection of respiratory and faecal samples did not have any direct influence on the differences in the CT values. This suggests that treatment and use of sewage for environmental surveillance of SARS COV-2 could be a potential public health countermeasure.

**Supplementary Information:**

The online version contains supplementary material available at 10.1186/s13104-024-06790-z.

## Introduction

Sewage and wastewater are important environmental samples for public health surveillance. Hence, establishing the presence of pathogens in such samples is important. Although COVID-19 is respiratory disease, the disease has been reported to affect other organ systems, making it a multisystem disease [1, 2]. A wide spectrum of symptoms have since been described to be associated with the infection. Gastrointestinal (GI) symptoms associated with COVID-19 include diarrhoea, abdominal pain, nausea, anorexia and vomiting [1–3]. The involvement of the GI system suggests the possible shedding in faeces, which may have implications for transmission via the faecal contamination routes.

Detection of SARS-CoV-2 in faecal samples is not new. In February 2020, scientists from the Laboratory of the National Institute for Viral Disease Control and Prevention isolated a 2019 novel coronavirus (2019 nCoV) strain from a stool specimen of a laboratory-confirmed COVID-19 severe pneumonia case [4]. Similarly, other studies have reported detection of SARS-CoV-2 faecal shedding in up to 50% of cases [5–7].

Generally, public health efforts to contain the spread of SARS-CoV-2 was focused on respiratory droplets and the respiratory route of transmission. However, the shedding of SARS-CoV-2 in faecal specimens makes it important for a rethink of the question of inadvertent human-to-human transmission via the faecal route and the exploitation of faecal materials for detection of the SARS-CoV-2 pathogen. It will therefore be useful to have adequate data and an understanding of the involvement of the GI tract in the transmission and diagnosis of COVID-19 in future pandemics.

## Materials and methods

### Patients, samples and data collection

Between July and August 2021, we enrolled 48 COVID-19 patients with laboratory-confirmed SARS-CoV-2 infection at the Ho Municipal Hospital and the University of Health and Allied Sciences (UHAS) COVID-19 Centre. The enrolment was done using convenient sampling. Briefly, patients who tested positive either at the treatment centre or from the lab walk-in services were approached and invited to participate by the study team. Those who consented were provided with stool containers for stool sample collection. Participants were guided on how to collect stool without contaminating them. Briefly, the stool was to be deposited on toilet tissue and then a small portion was picked in the centre using the spatula from the stool container without touching any other surface. Respiratory samples were taken from participants using nasopharyngeal swabs and placed in virus transport medium (VTM). The body temperature was also taken for each participant. The Ghana Health Service COVID-19 Case Base Forms were used to collect clinical and epidemiological data.

### SARS-CoV-2 RNA extraction and detection by real-time RT-PCR

Real-time reverse-transcriptase polymerase chain reaction (RT-PCR) was performed at the UHAS COVID-19 Centre. SARS-CoV-2 RNA was extracted from stool samples using the FavorPrep™ Viral Nucleic Acid Extraction Kit (Favorgen Biotech Corp., Taiwan) while from the nasopharyngeal swabs, SARS-CoV-2 RNA was obtained with the LBP nucleic acid extraction kit (Guangzhou, China). In both cases, the manufacturer’s instructions were strictly followed.

### RT-PCR for SARS-CoV-2 detection

The Novel Coronavirus (2019-nCoV) Nucleic Acid Diagnostic Kit (Sansure Biotech, China) was used for the detection of SARS-CoV-2 *ORF1ab* and *N* genes from the extracted RNA samples. The kit is a PCR probe-based test reported to have a sensitivity of 200 copies/ml. RT-PCR was done in 30 µL volume consisting of 26 µL qPCR mix and 4 µL of the extracted RNA sample. The amplification was done on the AGS4800 RT-PCR detection system (AGS Bioanyu, China) for cDNA synthesis at 55 °C for 3 min and 95 °C for 30 s and 45 cycles of quantification at 95 °C for 3 s and 60 °C for 12 s. A cycle threshold (CT) value between less or equal to 40 was considered positive according to the manufacturer. Kit positive and negative controls were included in the reaction set-up. Additionally, a previously tested positive sample and nuclease free water were always included through the extraction process to RT-PCR to serve as in-house positive and negative controls.

### Statistical analysis

Prevalence of SARS-CoV-2 was determined by simple proportion. Central tendency was estimated as means with standard deviations at 95% confidence intervals. Paired t-tests were used to compare CT values of the same gene between the faecal and nasopharyngeal samples. Unpaired t-test was used to test for differences between the CT values of genes in symptomatic and asymptomatic patients. Significance was determined at *P* < 0.05. GraphPad Prism 9 (GraphPad Software LLC, USA) was used for statistical analysis.

## Results

### Demographic and patient characteristics

A total of forty-eight (48) patients consented and were included in the study. Of these, 68.8% were females and the rest identified as males. Majority of the participants (60.4%) were asymptomatic whereas 39.6% were symptomatic. The symptoms reported by the study participants were headache (57.9%), weakness (57.9%), cough (52.6%), blocked/runny nose (47.4%), fever (31.6%), sore throat (31.6%), and shortness of breath (21.1%). None of the symptomatic patients complained of irritability or gastrointestinal (GI) symptoms (diarrhoea and vomiting) except one who complained of nausea (5.3%) (Table 1).

**Table 1:** Clinical information of study participants

Variable	Frequency	Percentage (%)
Infection outcome		
Symptomatic	22	45.8
Asymptomatic	26	54.2
Clinical manifestations		
Fever	6	31.6
Weakness	11	57.9
Cough	10	52.6
Sore throat	6	31.6
Shortness of breath	4	21.1
Nausea	1	5.3
Headache	11	57.9
Runny nose	8	42.1
Blocked nose	2	10.5
Anosmia	2	10.5

### Association of ORF1ab and N genes with SARS-CoV-2 infection statuses

The mean age for the symptomatic patients was 31 years whereas that of the asymptomatic patients was 28 years (Median 28 years). Average temperature for symptomatic and asymptomatic participants was 37 °C and 36 °C respectively. Further details on the demographic characteristics are provided in Table 2. There was no statistical difference between the CT values when compared for each gene between symptomatic and asymptomatic patients in both nasopharyngeal samples. For the faecal samples however, there was significant differences between the CT values obtained for the *ORF1ab* gene (p = 0.0031) and the *N* gene (p = 0.0005), where higher CT values were recorded in symptomatic participants. CT values and other raw data have been included as a Additional file 1.

**Table 2:** Descriptive values for demographic and laboratory data

Variable	Frequency (%)/Mean ± SD [95% CI]			P value
	Symptomatic	P value	Asymptomatic	
Number of participants	19 (39.58%)		29 (60.42%)	
Age; years	31 ± 13 (95% CI = 25–38)		28 ± 8.4 (95% CI = 23–31)	
Temperature; °C	37 ± 0.41 (95% CI = 37)		36 ± 0.36 (95% CI = 36–37)	
Faecal detection of SARS-CoV-2	18 (94.7%)		27 (93.1%)	0.9672*
Naso ORF1ab	30 ± 3.70 (95% CI = 29–32)	0.0094**	31 ± 5.5 (95% CI = 29–33)	0.0047**
Faecal ORF1ab	28 ± 0.35 (95% CI = 28)		26 ± 8.9 (95% CI = 22–29)	
Naso N gene	29 ± 4.2 (95% CI = 27–31)	0.0827**	30 ± 3.8 (95% CI = 28–31)	0.0072**
Faecal N gene	27 ± 0.54 (95% CI = 27)		25 ± 8.7 (95% CI = 22–28)	

^*^Chi square test

^**^Unpaired t-tests

### Shedding of SARS-CoV-2 in faeces

**Fig. 1:**
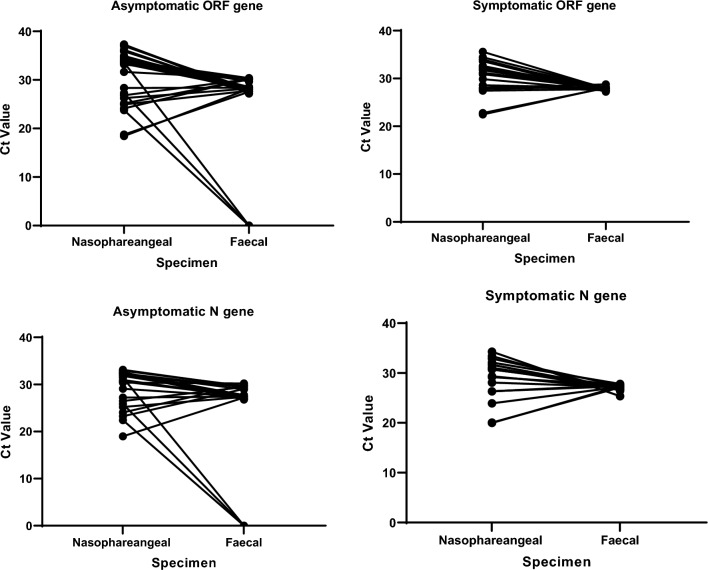
Differences between Ct values obtained from nasopharyngeal and faecal samples

Faecal shedding was observed in both symptomatic and asymptomatic patients. In the symptomatic patients, there was little variation in the CT values for both the *ORF1ab* (CV = 1.2%) and *N* genes (CV = 2.0%) whereas in the nasopharyngeal samples the variations were higher (12% for *ORF1ab* gene and 15% for *N* gene) (Fig. 1). The opposite was seen in the asymptomatic group where variation in the CT values for the faecal samples was higher (35% for both genes) and lower, 18% and 13% for nasopharyngeal *ORF1ab* and *N* genes respectively (Fig. 1). In both genes, the mean CT values were lower (suggesting higher viral loads) for the faecal samples compared to the nasopharyngeal samples (Table 1) for both the symptomatic and asymptomatic patients. The mean CT values of the genes in the faecal samples were lowest for both genes in the asymptomatic group.

## Discussion

This study reports viral shedding of SARS-CoV-2 in individuals confirmed with infection using a respiratory specimen, irrespective of the demographic and the symptomatic status. In several studies across the globe, faecal viral shedding in COVID-19 individuals have been reported [6, 8–12]. Evidence of faecal viral shedding in patients with COVID-19 is of public health importance. In addition to acquiring infections through respiratory droplets, people may also become infected through non-respiratory routes [13]. In areas where sanitation and hygiene are compromised, faecal contamination of surfaces could drive disease transmission. Viral shedding has been reported to occur longer before COVID-19 symptoms appear [14] and could last as long as 30 days after cessation of symptoms [15]. Furthermore, SARS-CoV-2 shedding time has also been found to be longer in faecal specimens than in respiratory specimens [15]. It can then be inferred that, COVID-19 patients discharged or de-isolated based on respiratory sample PCR negativity may pose high risk to another person living together. Furthermore, sewage could be a potential source of infection and for environmental surveillance.

Most of the study participants were asymptomatic which was a feature of the disease that presented a challenge to control measures. Only one patient reported nausea, which is a gastro-related symptom. It would have been expected that, the presence of the virus in the GI tract will cause a perturbation leading to one or more GI symptoms but this was not the case. Our finding is similar to other studies where about 67% of the study participants who demonstrated ongoing faecal shedding of SARS-CoV-2 presented with no GI symptoms [16–19]. Furthermore, a review of 48 studies on shedding of SARS-CoV-2 in faeces, severe gastrointestinal dysfunction was reported only in few COVID-19 cases. This included about 11 ± 2% who presented with diarrhea and 12 ± 3% who presented vomiting and nausea. It was also found that shedding of the virus in faeces peaked in the symptomatic period and persisted for several weeks although abundances declined when symptoms ceased [7]. Faecal samples can therefore be targeted as potential samples for investigations. Therefore, in future pandemics of similar biological nature, contacts traced may be screened using stool in addition to other samples.

This study reported that faecal samples could be a potential alternative to oro- or nasopharyngeal samples in the detection of SARS-CoV-2. This however requires further studies to establish detection limits and sensitive methods for viral nucleic acid isolation. In both symptomatic and asymptomatic cases, the CT values were found to be lower in faecal samples compared to corresponding respiratory samples. Nasopharyngeal swabs remain sensitive for collection of respiratory samples for SARS-CoV-2 detection in suspected cases. However, the sampling technique has a number of drawbacks [20], necessitating the evaluation of other non-invasive samples. The nasopharyngeal sampling can be challenging to obtain, especially by untrained and partially trained personnel. Additionally, collection of desirable quantity of specimens remains a challenge [21]. Further, nasopharyngeal swabbing causes discomfort and frequent reflex sneezing or coughing, hence, requires high-level personal protective equipment for healthcare workers, which are in short supply [22]. For public health countermeasures like surveillance and testing, faecal specimens are potential samples.

## Conclusion

In conclusion, this study found high viral shedding of SARS-CoV-2 in patients symptomatic for respiratory illness with or without gastrointestinal symptoms. It also emphasizes the importance of considering faecal shedding of SARS-CoV-2 as a potential route of transmission and the use of faecal samples and sewage for surveillance.

### Limitations

A limitation to the study is however the low number of samples but this was due to the pandemic at the time and challenges associated with recruiting willing patients. Furthermore, PCR is generally known to pick up some false positives. Although we assessed each positive and run independently against the controls that were included, the method cannot differentiate whether the viral particles are active or dead.

### Supplementary Information

**Additional file 1.** Study data.

## Data Availability

All study data collected in this study are presented in this publication.

## Abbreviations

COVID-19: Corona virus disease 2019
CT: Cycle threshold
GI: Gastrointestinal
RT-PCR: Real-time reverse-transcriptase polymerase chain reaction
SARS-CoV-2: Severe acute respiratory syndrome coronavirus 2
UHAS: University of Health and Allied Sciences
